# Depression in Persons with Diabetes by Age and Antidiabetic Treatment: A Cross-Sectional Analysis with Data from the Hordaland Health Study

**DOI:** 10.1371/journal.pone.0127161

**Published:** 2015-05-26

**Authors:** Line I. Berge, Trond Riise, Grethe S. Tell, Marjolein M. Iversen, Truls Østbye, Anders Lund, Ann Kristin Knudsen

**Affiliations:** 1 Department of Global Public Health and Primary Care, University of Bergen, Bergen, Norway; 2 Department of Clinical Medicine 1, Section of Psychiatry, University of Bergen, Bergen, Norway; 3 Mood Net Research Group, Haukeland University Hospital/University of Bergen, Bergen, Norway; 4 Centre for Evidence-Based Practice, Bergen University College, Bergen, Norway; 5 Section of Endocrinology, Department of Medicine, Stavanger University Hospital, Stavanger, Norway; 6 Global Health Institute, Duke University, North Carolina, United States of America; 7 Department of Health Registries, Norwegian Institute of Public Health, Bergen, Norway; University of Perugia, ITALY

## Abstract

**Background:**

Persons with diabetes have increased risk of depression, however, studies addressing whether the risk varies by age and type of antidiabetic treatment have yielded conflicting results. The aim of this study was to investigate if the association between diabetes and depression varied by type of antidiabetic treatment in a large community based sample of middle-aged (40–47 years) and older adults (70–72 years).

**Methods:**

Data from 21845 participants in the Hordaland Health Study (HUSK) were analyzed in a cross-sectional design. Diabetes was assessed by self-report and classified as un-medicated, treated by oral antidiabetic agents or by insulin. Depression was defined as a score ≥8 on the depression subscale of the Hospital Anxiety and Depression Scale and/or self-reported use of antidepressant agents. Associations between diabetes and depression were estimated using logistic regression.

**Results:**

Persons in their forties with diabetes had a doubled prevalence of depression (OR: 1.96 (95% C.I.: 1.35, 2.83)) compared to persons without diabetes, while a lower and non-significant association was found among persons in their seventies. Persons in their forties with orally treated diabetes had about three times higher prevalence of depression (OR: 2.92 (95% C.I.: 1.48, 5.77)) after adjustment for gender, BMI, physical activity, alcohol consumption and education, compared to non-diabetic persons in the same age-group. No association between depression and insulin or un-medicated diabetes was found.

**Conclusions:**

Clinicians should be aware that persons in their forties with orally treated diabetes are at a marked increased risk of depression.

## Introduction

Both depression and diabetes are prevalent and chronic diseases which negatively impact quality of life. The World Mental Health study reported a lifetime prevalence of major depressive disorders of about 15% in high-income countries [1], while the International Diabetes Federation estimates the prevalence of diabetes worldwide to exceed 8% [2]. Major depressive disorder is the third leading cause of disability adjusted life years (DALYS) in Norway, while diabetes is ranked as the seventh most important cause [3]. Both symptoms of depression and depressive disorders are prevalent among persons with diabetes. Compared to the non-diabetic population, depression has been found to be about twice as prevalent in individuals with both type 1 [4] and type 2 [5] diabetes. The World Mental Health Survey assessing psychiatric diagnoses using a structured diagnostic interview found an OR of 1.38 (95% CI: 1.15, 1.66) for major depression among persons with diabetes compared to persons without diabetes in an unselected worldwide sample of about 85000 persons [6].

It is well established that comorbid depression increases the risk of adverse outcomes among persons with diabetes. Compared to persons with only diabetes, those with both diabetes and depression have poorer quality of life [7], self-care and adherence to medical regimens [8–9], higher risk of complications [10] and 50% increased mortality [11]. Despite the high prevalence and great impact of comorbid depression in persons with diabetes, few studies have addressed whether the risk of depression varies by type of treatment, and the results are conflicting [12–14]. Increased risk of depression among persons with type 2 diabetes has been found exclusively for those using antidiabetic agents [12], for those not using antidiabetic agents [14] and irrespective of use of antidiabetic agents [13,15]. Further, a recent systematic review of the epidemiology of depression and diabetes concludes that the relationship between age and risk of depression in people with diabetes remains complex and needs further investigation [16]. While information on how these diseases coexist in the population is desirable to help clinicians detect and target interventions for depression among individuals with diabetes, some argue that exploration of psychiatric-somatic comorbidity also contribute to improved understanding of the pathophysiology and biologic treatment of psychiatric disorders [17].

The aim of this study was to investigate if the association between diabetes and depression varied by type of antidiabetic treatment in a large community based sample of middle-aged and older adults.

## Materials and Methods

### Participants and data collection

Data in the present study were derived from the community based Hordaland Health Study (HUSK) conducted in western Norway during 1997–1999. HUSK was a collaboration between the National Health Screening Service, the University of Bergen and local health services. All individuals born 1953–57 who resided in Hordaland County on December 31, 1997 were invited to participate (N = 29400). In addition, 4849 persons born 1950–51 and 4338 persons born 1925–27, who had participated in a previous health study (the Hordaland Homocysteine study) in 1992–1993, were invited. While 63% of persons born 1953–57 participated, the participation rate in both the 1950–1951 and 1925–27 cohorts were 77%. The final sample of eligible HUSK participants consisted of 25532 persons.

Data collection was conducted in three steps, consisting of two sets of questionnaires and one brief physical health examination. The first questionnaire was included with a mailed personal invitation to take part in the study. This questionnaire was returned at the health examination, at which height and weight were measured. Participants were then given a second questionnaire to be returned by mail in a prepaid envelope.

Included in the present study were persons who attended the examination and completed both questionnaires. Persons without valid responses on questions about symptoms of depression defined as responding to 4 or fewer of the 7 items included in the depression subscale on the Hospital Anxiety and Depression Scale (HADS-d), were excluded (n = 3671 (14.4%)). We further excluded 16 persons (0.1%) who reported a history of all six somatic conditions assessed in the first questionnaire (infarction, angina, stroke, diabetes, asthma and multiple sclerosis), as these persons were assumed to have misinterpreted the form. The final analysis sample for the present study consisted of 21845 persons, 85.6% of the total number of HUSK participants. Of these, 18948 were 40–47 years and 2897 were 70–72 years.

### Measures

#### Diabetes

Presence of diabetes was assessed with the item “have you, or have you had diabetes”. Persons answering affirmative were further classified according to type of antidiabetic treatment as 1) un-medicated diabetes (no use of antidiabetic agents), 2) orally treated diabetes (with or without use of insulin) and 3) insulin treated diabetes (and no oral antidiabetic agents). Antidiabetic agents were defined according to the 1997 anatomical therapeutic chemical (ATC) classification system and included all agents categorized under A10A (insulins), A10B (metformin, glibenklamid, klorpropramid, blipizid and glucobay) and AX2 (glimeperid). The sensitivity and specificity of self-reported diabetes has been shown to range from 58.5% to 70.8% and 95.6% to 96.8% respectively, depending on the diagnostic criteria applied for diabetes [18].

#### Depression

Two measures of depression were used in the present study: 1) symptoms of depression during the last week assessed by HADS-d and 2) self-reported use of antidepressant agents the day before completing the first questionnaire. HADS consists of 14 four-point Likert-scaled items, 7 measuring symptoms of depression (HADS-d) and 7 measuring anxiety (HADS-a). A higher score indicates a higher symptom burden. HADS was originally designed for symptom screening in hospital settings, and excludes items that may be attributed to somatic illness to reduce the likelihood of false-positive cases among individuals with somatic diseases. HADS is also considered a valid case-finding instrument of both depression and anxiety in the general population [19]. HADS-d was used as a dichotomous variable with cut-off level of ≥8 for “caseness of depression”, which has been shown to yield a sensitivity and specificity of about 0.8 each [19]. To avoid misclassification of persons with recent depression now in remission due to treatment, we also classified persons reporting use of antidepressant agents the day before completing the questionnaire as depressed. Antidepressant agents were defined according to the 1997 ATC-classification system, and encompassed all agents categorized under N06A (including tricyclic and tetracyclic antidepressant agents, SSRI) and NX5 (SNRI).

Three depression variables were computed based on the two depression measures: 1) HADS-d ≥8 (reference group: HADS-d<8), 2) use of antidepressant agents (reference group: no use of antidepressant agents) and 3) HADS-d ≥8 and/or use of antidepressant agents (reference group: HADS-d<8 and no use of antidepressant agents).

#### Possible confounders

For a factor to be a confounder in the association between diabetes and depression, it needs to be associated with both conditions and further not expected to be on the causal pathway between them. Based on previous knowledge [13,20–21], we *a priori* selected musculoskeletal pain, smoking, body mass index (BMI), physical activity, alcohol consumption, education and cohabiting as possible confounders. Weight (in kilograms) and height (in meters) were measured at the health examination, while self-reported information on other variables included in this study was obtained from the questionnaires. Musculoskeletal pain was defined as a history of painful and/or stiff muscles or joints of at least 3 months duration during the last 12 months. Smoking was categorized as “never”, “former” and “current”. Body mass index (BMI) was calculated as weight (kg)/height (m)^2^, and categorized as underweight (≤19.9), normal, (20.0–24.9), overweight (25.0–29.9) and obese (≥30). Information on light physical activity (no sweating or getting out of breath) and hard physical activity (sweating or getting out of breath) was reported as hours per week in four groups (none, ≤1, 1–2, and ≥ 3). No physical activity was given the value 1, ≤1hour value 2, 1–2 hours value 3, and ≥ 3 value 4, and a summary score of physical activity was computed multiplying the value of hard physical activity by two, and adding light physical activity, yielding a continuous score ranging from 3–12. Alcohol consumption was defined as number of alcoholic units consumed per fortnight and categorized as ≤1, 2–5 and ≥6. Cohabiting was defined as being married or living with a partner, as opposed to being unmarried, widowed, separated or divorced. Highest achieved education was categorized as compulsory school only (up to ten years), high school and higher education (college or university).

### Statistical methods

A total of 220 (1.0%) persons had missing responses on the question assessing diabetes. These were classified as not having diabetes. A total of 128 (0.6%) persons had valid responses on 5 of the 7 items on HADS-d, while 2064 (8.3%) had valid responses on 6 of the 7 items. These persons were given imputed values based on the mean value of the non-missing responses. Missing values on any of the confounding factors were handled as follows in the final regression analysis: 1) imputed mean value on physical activity if valid response on the other item regarding this topic (n = 784 (4.1%)), 2) missing as separate category on variable alcohol consumption (n = 496 (2.6%)), and 3) exclusion of cases with otherwise missing values (n = 321 (1.7%)).

**Fig 1:**
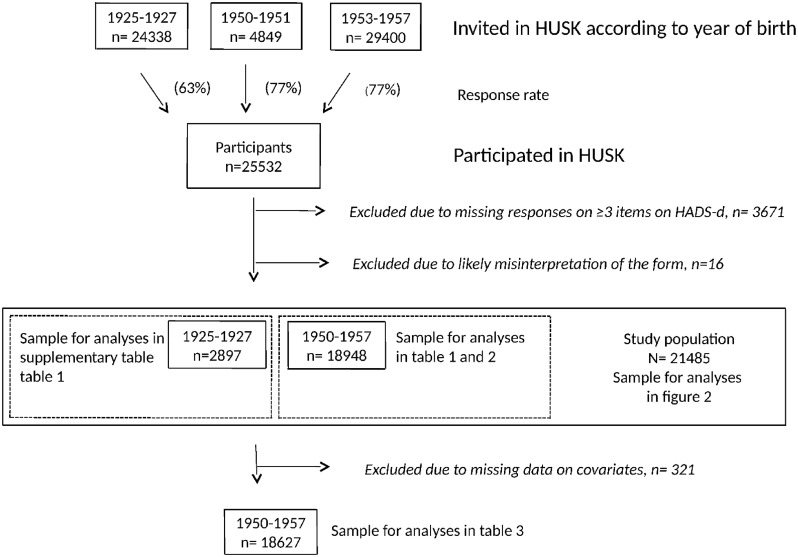
Flow chart. Inclusion and exclusion procedures in the Hordaland Health Study (HUSK), and study samples included in the various analyses.

Sample characteristics were examined with descriptive statistics. Logistic regression analyses were used in a cross-sectional design to examine the association between diabetes (independent variable) and depression (dependent variable). Effect estimates were given as OR with 95% confidence intervals. Stratified analyses by age groups were performed to examine the effect of diabetes on the three measures of depression. To test for possible differences in the effect between age groups, we included an interaction term between age group and diabetes in the model. We then examined the association between the three different types of antidiabetic treatment and the three depression variables among persons aged 40–47 years and 70–72 years. For persons aged 40–47 years, we conducted a sensitivity analysis examining the associations between the three depression variables and antidiabetic treatment further categorizing orally treated diabetes in orally treated diabetes monotherapy and insulin and orally treated diabetes. Presence of a statistical significant association between both diabetes and depression (HADS-d ≥8 and/or use of antidepressant agents yesterday) were examined for the covariates selected a priori for persons aged 40–47 years. Covariates with statistically significant associations with both diabetes and depression were considered confounders and included in the final model when examining the association between the three different types of antidiabetic treatment and depression defined as HADS-d ≥8 and/or use of antidepressant agents among persons aged 40–47 years. A flow chart illustrating inclusion and exclusion criteria and the study samples included in various analyses are presented in Fig 1. All statistical analyses were performed using SPSS version 20.

### Ethics

The study protocol was approved by the Regional Ethics Committee of Western Norway. Written informed consent was obtained from all participants at the time of the health examination.

## Results

**Fig 2:**
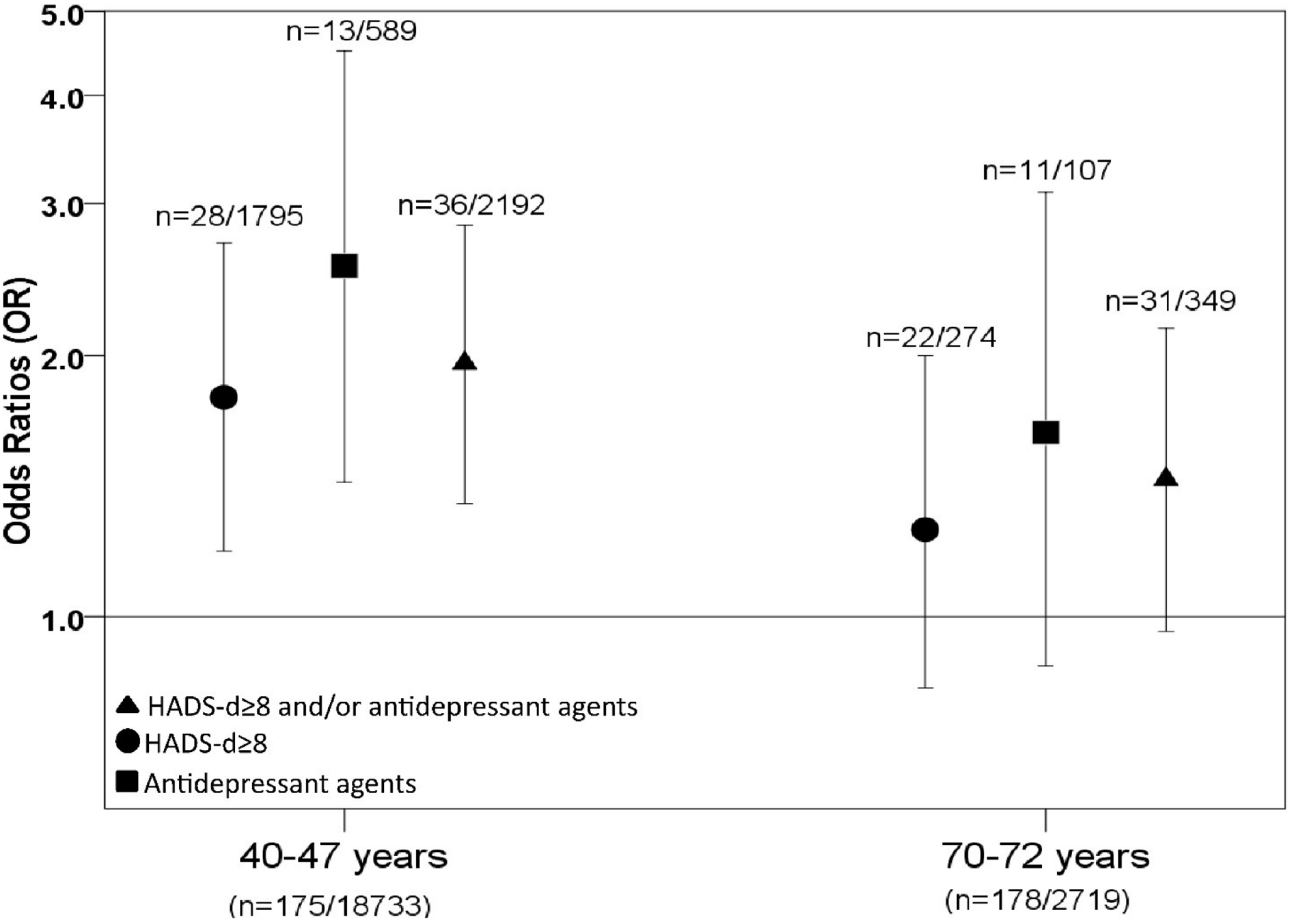
Associations between depression and diabetes by age in the Hordaland Health Study (HUSK). OR with 95% confidence intervals (vertical bars), adjusted for gender. Numbers in brackets represent number of persons with diabetes relative to the number without diabetes.

Considering both age-groups, 67 of 353 persons with diabetes (19.0%) reported HADS-d≥8 and/or use of antidepressant agents. Relative to the non-diabetic population, this gave an overall age and gender adjusted OR of 1.69 (95% CI: 1.28, 2.22). When stratifying by age-group, significant associations between diabetes and all 3 measures of depression were found for persons aged 40–47 years, while the associations between diabetes and any measure of depression in the age group 70–72 years were lower and not statistically significant (Fig 2). However, the differences in the OR’s between the two age groups were not significant when interaction terms were included in the model (p ≥ 0.05 for all measures, data not shown). Mean duration of diabetes was 15 years both for persons aged 40–47 years and 70–72 years.

As shown in Fig 2, OR for HADS-d≥8 and/or use of antidepressant agents for persons with diabetes in their forties was 1.96 (95% CI: 1.35, 2.83), while the corresponding OR for persons in their seventies was 1.44 (95% CI: 0.96, 2.15). Analyses stratified by gender revealed no gender differences in the outcome within the same age group. In the age-group 40–47 years, OR for men and women were 1.65 (95% CI: 0.94, 2.90), and 2.25 (95% CI: 1.28, 3.67) respectively (interaction term: p = 0.42). Among the 70–72 years the corresponding ORs were 1.58 (95% CI: 0.88, 2.83) for men and 1.32 (95% CI: 0.76, 2.31) for women (interaction term: p = 0.67).

No significant associations between any type of antidiabetic treatment and any measure of depression were found among persons aged 70–72 years (Table 1). Compared to those without diabetes, persons aged 40–47 years with un-medicated diabetes had an increased OR of 2.33 (95% CI: 1.07, 5.07) for use of antidepressant agents, while no association was found with HADS-d≥8 and/or use of antidepressant agents (Table 1). Oral treatment of diabetes was significantly associated with all three measures of depression, the strongest association was found with use of antidepressant agents with an OR of almost five. Conducting a sensitivity analysis by further categorizing persons aged 40–47 years using oral antidiabetic agents in those using oral antidiabetic agents in monotherapy and those using the combination of insulin and oral antidiabetic agents did not markedly change the results for the group using oral antidiabetic agents in monotherapy. As all three patients receiving the combination of insulin and oral antidiabetic agents reported HADS-d ≥8, no estimate could be calculated for this group (S1 Table). No significant associations with any measure of depression were found for insulin treated diabetes.

**Table 1:** Associations of various measures of depression by antidiabetic treatment for persons aged 40–47 years and 70–72 years in the Hordaland Health Study (HUSK).

40–47 years (n = 18948)	*HADS-d≥8*		*Antidepressant agents*		*HADS-d≥8 and/or antidepressant agents*	
	n (%)	OR (95% CI)*	n(%)	OR (95%CI)*	n(%)	OR (95%CI)*
*No diabetes* (n = 18773)	1795	1 (ref)	589	1 (ref)	2192	1 (ref)
	(9.6)		(3.1)		(11.7)	
*Un-medicated diabetes* (n = 97)	12	1.35 (0.74, 2.48)	7	2.33 (1.07, 5.07)	18	1.50 (0.87, 2.56)
	(12.4)		(7.2)		(16.5)	
*Orally treated diabetes* (n = 42)	10	2.86 (1.40, 5.83)	5	4.79 (1.86, 12.35)	14	3.76 (1.98, 7.16)
	(23.8)		(11.9)		(33.3)	
*Insulin treated diabetes* (n = 36)	6	1.87 (0.78, 4.50)	1	0.92 (0.13, 6.75)	6	1.51 (0.63, 3.63)
	(16.7)		(2.8)		(16.7)	
**70–72 years** (n = 2897)						
*No diabetes* (n = 2719)	274	1 (ref)	107	1 (ref)	349	1 (ref)
	(10.1)		(3.9)		(12.8)	
*Un-medicated diabetes* (n = 78)	11	1.47 (0.77, 2.81)	5	1.68 (0.66, 4.26)	15	1.62 (0.91, 2.88)
	(14.1)		(6.4)		(19.2)	
*Orally treated diabetes* (n = 87)	9	1.03 (0.51, 2.08)	5	1.51 (0.60, 3.81)	14	1.31 (0.73, 2.34)
	(10.3)		(5.7)		(16.1)	
*Insulin treated diabetes* (n = 13)	2	1.62 (0.36, 7.36)	1	2.05 (0.26, 15.96)	2	1.24 (0.27, 5.60)
	(15.4)		(7.7)		(15.4)	

*adjusted for gender

For persons aged 40–47 years, all the *a priori* identified potential confounding factors were associated with depression defined as HADS-d≥8 and/or use of antidepressant in the crude model (data not shown). As the distribution of musculoskeletal pain, smoking and cohabiting did not differ significantly by diabetes status (p-level of 0.05) (Table 2), only BMI, physical activity, alcohol consumption and education were regarded as confounders for the association between diabetes and depression, and included in the final model.

**Table 2:** Distribution of covariates by diabetes status for persons aged 40–47 years in the Hordaland Health Study (HUSK).

40–47 years (n = 18948)	*Diabetes (n = 175)*	*No diabetes (n = 18773)*	p-value*
	*N (%)*	*N (%)*	
*Sex (% women)*	93 (51.3)	10457 (55.2)	0.59
*Missing*	0 (0)	0 (0)	
*Muskuloskeletal pain*	87 (49.7)	8018 (42.7)	0.18
*Missing*	1 (0.6)	123 (0.7)	
*Smoking*			
No	62 (35.4)	6143 (32.7)	0.72
Former	52 (29.7)	5336 (28.4)	
Current	61 (34.9)	7276 (38.8)	
Missing	0 (0.0)	18 (0.1)	
*BMI*			
≤ 19.9	2 (1.1)	769 (4.1)	<0.01
20.0–24.9	46 (26.3)	8812 (46.9)	
25.0–29.9	62 (35.4)	7165 (38.2)	
≥30.0	65 (37.1)	2001 (10.7)	
Missing	0 (0.0)	26 (0.1)	
*Physical activity* ^*§*^	7.2 (6.8, 7.6)	7.7 (7.7, 7.8)	<0.01**
*Missing*	3 (1.7)	170 (0.9)	
*Alcohol units pr 14 days*			
≤1	85 (48.6)	6264 (33.4)	<0.01
2–5	38 (21.7)	5792 (30.9)	
≥6	40 (22.9)	6233 (33.2)	
Missing	12 (6.9)	484 (2.6)	
*Education*			
Less than high school (up to ten years)	51 (29.1)	3329 (17.7)	<0.01
High school	73 (41.7)	8488 (45.2)	
College or university	48 (27.4)	6805 (36.2)	
Missing	3 (1.7)	151 (0.8)	
*Cohabiting*	123 (70.3)	14036 (74.8)	0.18
*Missing*	0 (0)	0 (0)	

*Pearson Chi-square test

** Independent sample t-test

^§^Mean on a scale from 3–12 (95% CI)

Table 3 shows associations between diabetes and depression (HADS-d≥8 and/or use of antidepressant agents) stratified by antidiabetic treatment and with adjustment for the confounders, one by one and combined. BMI was associated with the strongest attenuation of the effect with a reduction of OR from 3.79 to 3.16 for orally treated diabetes and from 1.53 to 1.34 for un-medicated diabetes. After adjustment for all confounders, the association between orally treated diabetes and depression remained significant (OR 2.92 (95% CI: 1.48, 5.77)).

**Table 3:** Crude associations between depression and diabetes and depression and the confounders for persons aged 40–47 years, and associations between diabetes and depression adjusted for confounders in the Hordaland Health Study (HUSK).

40–47 years (n = 18627)	*No depression* (n = 16499)	*Depression* HADS-d≥8 and/or antidepressant agents (n = 2178) N (%)	Crude OR* (95% CI)	Adjusted for BMI* OR (95% CI)	Adjusted for physical activity* OR (95% CI)	Adjusted for alcohol consumption* OR (95% CI)	Adjusted for education* OR (95% CI)	Adjusted for BMI, physical activity, alcohol and education* OR (95% CI)
	*N (%)*							
*No diabetes* (ref)	16313 (99.2)	2144 (98.4)	1 (-)	1 (-)	1 (-)	1 (-)	1 (-)	1 (-)
*Un-medicated diabetes*	80 (0.5)	16 (0.7)	1.53 (0.89, 1.61)	1.34 (0.78, 2.30)	1.39 (0.81, 2.40)	1.43 (0.83, 2.45)	1.44 (0.84, 2.47)	1.18 (0.68, 2.04)
*Orally treated diabetes*	26 (0.2)	13 (0.6)	3.79 (1.94, 7.39)	3.16 (1.61, 6.20)	3.71 (1.89, 7.31)	3.42 (1.75, 6.69)	3.53 (1.80, 6.91)	2.92 (1.48, 5.77)
*Insulin treated diabetes*	30 (0.2)	5 (0.2)	1.26 (0.49, 3.26)	1.20 (0.46, 3.09)	1.21 (0.47, 3.15)	1.14 (0.44, 2.94)	1.18 (0.46, 3.05)	1.04 (0.40, 2.73)
*BMI*								
≤19.9	645 (3.9)	112 (5.1)	1.52 (1.23, 1.89)	1.53 (1.23, 1.89)				1.50 (1.21, 1.86)
20.0–24.9	7833 (47.6)	898 (41.2)	(-)	(-)				(-)
25.0–19.9	6280 (38.2)	828 (38.0)	1.14 (1.03, 1.27)	1.14 (1.03, 1.26)				1.09 (0.98. 1–21)
≥30	1691 (10.3)	340 (15.6)	1.75 (1.52, 2.00)	1.71 (1.50, 1.97)				1.47 (1.28, 1.69)
*Physical activity* Pr unit increase Highest level compared to lowest			0.86 (0.84, 0.88)		0.86 (0.84, 0.88)			0.88 (0.86, 00.89)
*Alcohol consumption* (units pr 14 days)								
≤1 (ref)	5313 (32.2)	878 (40.3)	(-)			(-)		(-)
2–5	5198 (31.6)	544 (25.0)	0.63 (0.56, 0.70)			0.63 (0.56, 0.71)		0.69 (0.61, 0.77)
≥6	5545 (33.7)	677 (31.1)	0.71 (0.64, 0.80)			0.72 (0.64, 0.80)		0.81 (0.72, 0.91)
Missing	393 (2.4)	79 (3.6)	1.21 (0.94, 1.56)			1.20 (0.94, 1.55)		1.22 (0.94, 1.57)
*Education*								
Less than high school	2790 (17.0)	536 (24.6)	(-)				(-)	(-)
High school	7481 (45.5)	1006 (46.2)	0.70 (0.62, 0.78)				0.70 (0.62, 0.78)	0.78 (0.69, 0.87)
College or university	6178 (37.6)	636 (29.2)	0.53 (0.47, 0.60)				0.54 (0.47, 0.61)	0.64 (0.57, 0.73)

*Adjusted for gender.

## Discussion

In the present study of a large sample of participants from the general population, we found that persons in their seventies with diabetes had little increased prevalence of depression, while those in their forties with diabetes had about twice as high prevalence of depression relative to persons without diabetes in the respective age group. Further, persons in their forties with un-medicated diabetes reported no increased prevalence of symptoms of depression, but nevertheless had approximately twice the prevalence of use of antidepressant agents compared to persons without diabetes. Persons in their forties with orally treated diabetes had about three and five times higher prevalence of depression measured by symptoms and use of antidepressant agents, respectively. We found that as much as one third of the persons using oral antidiabetic agents either reported symptoms of depression/and or using antidepressant agents. This is within the range of the estimated prevalence of depression among persons with diabetes type 2 in the recent systematic review of the epidemiology of depression and diabetes [16], and contrasts the corresponding proportion in the non-diabetic population of 12%. Finally, the association between diabetes and the combined measure of depression could not be fully explained by the confounding factors BMI, physical activity, alcohol consumption and education.

The main strength of the study is use of data from HUSK, a large, comprehensive and well conducted community based study. Important limitations are possible selection bias due to an overall participation rate of 66%, and self-reported information, as well as to low power to detect significant differences between age groups and types of treatment. Further, persons with severe psychiatric or somatic diseases are less likely to participate in health surveys [22], in particular, a higher prevalence of diabetes and psychiatric disorders was found among non-participants relative to participants in a large population based Norwegian study [23]. However, this is more likely to underestimate the prevalence estimates of the diseases rather than reducing the validity of measures of associations between the diseases [22]. Finally, when interpreting the associations adjusted for identified confounders, we cannot exclude bias due to residual confounding.

It has been argued that one should assess both symptoms of depression and use of antidepressant agents when estimating overall prevalence of depression to avoid possible misclassification of persons without elevated symptoms of depression as non-depressed due to effect of antidepressant treatment [24]. Even though antidepressant agents most often are prescribed for depression, it can also be used in treatment of anxiety, post-traumatic stress disorders, eating disorders and neuropathic pain, thereby reducing the specificity of our measure of depression. Nonetheless, we might misclassify persons with depression now in remission due to psychotherapeutic treatment, as we did not have information on whether participants had received this treatment. A recent systematic review on screening tools for depression among persons with diabetes found that HADS-d is frequently used in screening for depressive symptoms in diabetes [25]. The authors argued that due to the exclusion of items that could be confounded with symptoms of poorly regulated diabetes, HADS-d was likely more valid in diabetic populations than other commonly used screening tools for depression. Nevertheless, as with several of the other screening tools evaluated, HADS-d was found to give a relatively high rate of “false positive” cases thus possibly overestimating the prevalence of depression. If persons with diabetes are more likely than persons without diabetes to be misclassified as depressed, our estimates are likely higher than the true association.

A validation study from the Netherlands comparing self-reported information on different cardiovascular diseases and their risk factors with information from medical records concluded that self-reported information on diabetes is valid [26]. Nevertheless, the sensitivity of a self-reported diagnosis of diabetes is considered modest, ranging from about 60% to 70% [18]. If misclassification of persons with diabetes as non-diabetics is independent of caseness of depression, we likely underestimate the true association between diabetes and depression. Further, we did not have specific information on whether a person had type 1 or 2 diabetes. Still, we argue that as groups, those treated with insulin and no oral antidiabetic agents can be considered having type 1 diabetes, whereas those treated with oral antidiabetic agents, irrespective of use of insulin, and those not receiving medication can be assumed to have type 2 diabetes [27]. Other studies in the field of diabetes epidemiology have also relied on this assumption [28]. Nevertheless, the estimates of the association between depression and type of diabetes should be interpreted with caution.

Our results indicate that increased prevalence of depression for persons with diabetes is most evident for those treated with oral antidiabetic agents. This contrasts with the results from a comprehensive study from Taiwan using diagnostic codes from a national health insurance database in a rather complex prospective design. The study found an increased risk of depression among persons with type 2 diabetes not using medical treatment and no increased risk of depression among those treated with metformin and sulfonylurea [14]. The same research group has also shown related beneficial effects of medical treatment with regard to possible increased risk of both Parkinson’s disease and dementia among persons with type 2 diabetes. [29–30]. These beneficial effects on the risk of depression exclusively among persons treated with oral antidiabetic agents are in sharp contrast with results from other studies. The cross-sectional Multiethnic Study of Atherosclerosis found increased symptoms of depression to be associated with treated type 2 diabetes (OR crude model: 1.57 (95% C.I.: 1.27, 1.96)), while no association was found with both un-medicated diabetes and with impaired glucose tolerance [12]. Finally, the Nurses’ Health Study examined the association in a prospective design, finding that persons with dietary and orally treated diabetes had an approximate similar increased risk of depression of about 40% [13], while the prevalence of both major and minor depression were fairly equal among persons with dietary and orally treated diabetes at baseline in the Pathways Epidemiologic Study [15]. The risk of depression according to type of treatment for diabetes remains complex, and future studies should address this concern.

Most studies investigating the risk of depression among persons with diabetes provide age-adjusted estimates, thus concealing potential variations in risk according to age. Other studies have evaluated the effect of age more indirectly. In a mixed sample of diabetes type 1 and 2 in Ireland, older age was found to be protective of symptoms of depression [31], and the Path Through Life Study comparing risk factors for depression in cohorts of persons in their forties and sixties found presence of diabetes to predict depressive symptoms only among those in their forties [32]. Covering the complete Norwegian population using medication as a proxy for disease, we have previously shown that the risk of depression when using oral antidiabetic agents is highest for persons in their thirties and markedly decreases with increasing age [27]. Moreover, a community based study of approximately 500 persons with type 2 diabetes aged 21 to 80 years found younger age to be independently associated with higher levels of depressed affect [33], while a study using data from the Behavioral Risk Factor Surveillance System showed that the prevalence of both major depression, major and minor depression and symptoms of depression were highest in age group 30–39 and 40–49 [34]. Further, a study using diagnostic codes from a national health insurance database in Taiwan found that the relative risk of incident depression in persons with diabetes, relative to the non-diabetic group, was highest among those less than 35 years [35]. Consistent with these observations, the findings from the present study show that younger age is a risk factor for depression in persons with diabetes. The lower risk of depression among older persons with diabetes relative to those younger could be attributed to psychological mechanisms, such as increasing experience with how to manage and cope with illnesses with age. Additionally, the reference group consisting of persons without diabetes may have higher prevalence of other chronic diseases in the oldest age group, thus reducing the possible “stigma” associated with having a chronic disease such as diabetes at older ages.

We can further only speculate why persons in their forties using oral antidiabetic agents have increased prevalence of depression, even after adjustments for BMI, physical activity, alcohol consumption and level of education. One possible explanation is that treatment with oral antidiabetic agents itself can cause depression; however, we find no support for this in the literature. Second, as we did not have information on level of HbA1c in this study, one possible proxy for severity of the disease is type of antidiabetic treatment. If we assume that those treated with oral antidiabetic agents have a disease that is more difficult to treat than those who successfully manage their diabetes with lifestyle interventions, one might argue that persons with a more difficult to regulate diabetes have a higher risk of depression compared to those with easier manageable diabetes. This is in agreement with previous studies finding depression to be associated with higher levels of HbA1c [36]. Further, there is convincing evidence that the association between diabetes type 2 and depression is bidirectional, and that the association is even stronger when considering depression as the exposure and diabetes as the outcome in prospective studies [13,35,37]. If we assume that persons with depression are more frequently seen by physicians, they might be more likely to be diagnosed with diabetes compared to persons without depression, particularly since it is estimated that up to 50% of diabetes cases may be undiagnosed [2]. Moreover, when persons struggling with depression are diagnosed with type 2 diabetes, they might be more likely to receive medical treatment if the physician decides that change of lifestyle is too difficult to achieve, possibly explaining the increased prevalence of depression only among those with diabetes treated with oral antidiabetic agents. In addition, one recent meta-analysis and one systematic review conclude that use of antidepressant agents is associated with increased risk of incident type 2 diabetes [38–39]. This is in agreement with our finding of an association between un-medicated diabetes and use of antidepressant agents, and that the strongest association found in our study was between orally treated diabetes and use of antidepressant agents.

Finally, one could hypothesize that younger age is a risk factor for depression among persons with diabetes due to shared etiology. Increased incidence of diabetes type 2 has been found for persons with low socioeconomic status, an association partly mediated by low grade inflammation [40]. If persons exposed to psychological stressors are more prone to develop chronic diseases at an earlier age [41], it might explain why younger persons with type 2 diabetes have higher risk of depression, poorer glycemic control and self-reported health compared to older persons with diabetes [42].

This study has limitations related to the cross-sectional design, the relatively low number of cases and lack of clinical data such HbA1c and number of complications. Nevertheless, the result points to the need of future prospective studies aiming at identifying factors associated with the increased occurrence of depression among young persons with diabetes treated with oral antidiabetic agents. From a public health perspective, it is important to target possible preventive strategies to reduce the prevalence and impact of depression comorbid to diabetes.

## Supporting Information

S1 Table Associations of various measures of depression by antidiabetic treatment for persons aged 40–47 years in the Hordaland Health Study operationalizing diabetes as a 5 category variable. (DOCX)

## References

[1] Bromet E, Andrade LH, Hwang I, Sampson NA, Alonso J, de Girolamo G, et al (2011) Cross-national epidemiology of DSM-IV major depressive episode. BMC Med 9: 90 10.1186/1741-7015-9-90 21791035 PMC3163615

[2] International Diabetes Federation (2013) Diabetes Atlas, sixth edition.

[3] Institute for Health Metrics and Evaluation (2013) The global burden of disease: Generating Evidence, Guiding Policy European Union and European Free Trade Association Regional Edition. Seattle, WA:, University of Washington.

[4] Gendelman N, Snell-Bergeon JK, McFann K, Kinney G, Paul Wadwa R, Bishop F, et al (2009) Prevalence and correlates of depression in individuals with and without type 1 diabetes. Diabetes Care 32: 575–579. 10.2337/dc08-1835 19171719 PMC2660458

[5] Ali S, Stone MA, Peters JL, Davies MJ, Khunti K (2006) The prevalence of co-morbid depression in adults with Type 2 diabetes: a systematic review and meta-analysis. Diabet Med 23: 1165–1173. 17054590 10.1111/j.1464-5491.2006.01943.x

[6] Lin EH, Korff MV, Alonso J, Angermeyer MC, Anthony J, Bromet E, et al (2008) Mental disorders among persons with diabetes—results from the World Mental Health Surveys. J Psychosom Res 65: 571–580. 10.1016/j.jpsychores.2008.06.007 19027447 PMC3672403

[7] Schram MT, Baan CA, Pouwer F (2009) Depression and quality of life in patients with diabetes: a systematic review from the European depression in diabetes (EDID) research consortium. Curr Diabetes Rev 5: 112–119. 19442096 10.2174/157339909788166828 PMC2764861

[8] Gonzalez JS, Peyrot M, McCarl LA, Collins EM, Serpa L, Mimiaga MJ, et al (2008) Depression and diabetes treatment nonadherence: a meta-analysis. Diabetes Care 31: 2398–2403. 10.2337/dc08-1341 19033420 PMC2584202

[9] Gonzalez JS, Safren SA, Cagliero E, Wexler DJ, Delahanty L, Wittenberg E, et al (2007) Depression, self-care, and medication adherence in type 2 diabetes: relationships across the full range of symptom severity. Diabetes Care 30: 2222–2227. 17536067 10.2337/dc07-0158 PMC4440862

[10] Lin EH, Rutter CM, Katon W, Heckbert SR, Ciechanowski P, Oliver MM, et al (2010) Depression and advanced complications of diabetes: a prospective cohort study. Diabetes Care 33: 264–269. 10.2337/dc09-1068 19933989 PMC2809260

[11] Park M, Katon WJ, Wolf FM (2013) Depression and risk of mortality in individuals with diabetes: a meta-analysis and systematic review. Gen Hosp Psychiatry 35: 217–225. 10.1016/j.genhosppsych.2013.01.006 23415577 PMC3644308

[12] Golden SH, Lee HB, Schreiner PJ, Diez Roux A, Fitzpatrick AL, Szklo M, et al (2007) Depression and type 2 diabetes mellitus: the multiethnic study of atherosclerosis. Psychosom Med 69: 529–536. 17636146 10.1097/PSY.0b013e3180f61c5c

[13] Pan A, Lucas M, Sun Q, van Dam RM, Franco OH, Manson JE, et al (2010) Bidirectional association between depression and type 2 diabetes mellitus in women. Arch Intern Med 170: 1884–1891. 10.1001/archinternmed.2010.356 21098346 PMC3065781

[14] Wahlqvist ML, Lee MS, Chuang SY, Hsu CC, Tsai HN, Yu SH, et al (2012) Increased risk of affective disorders in type 2 diabetes is minimized by sulfonylurea and metformin combination: a population-based cohort study. BMC Med 10: 150 10.1186/1741-7015-10-150 23194378 PMC3529194

[15] Heckbert SR, Rutter CM, Oliver M, Williams LH, Ciechanowski P, Lin EH, et al (2010) Depression in relation to long-term control of glycemia, blood pressure, and lipids in patients with diabetes. J Gen Intern Med 25: 524–529. 10.1007/s11606-010-1272-6 20182815 PMC2869429

[16] Roy T, Lloyd CE (2012) Epidemiology of depression and diabetes: a systematic review. J Affect Disord 142 Suppl: S8–21. 10.1016/S0165-0327(12)70004-6 23062861

[17] Schumann G, Binder EB, Holte A, de Kloet ER, Oedegaard KJ, Robbins TW, et al (2014) Stratified medicine for mental disorders. Eur Neuropsychopharmacol 24: 5–50. 10.1016/j.euroneuro.2013.09.010 24176673

[18] Schneider AL, Pankow JS, Heiss G, Selvin E (2012) Validity and reliability of self-reported diabetes in the atherosclerosis risk in communities study. Am J Epidemiol 176: 738–743. 10.1093/aje/kws156 23013620 PMC3571247

[19] Bjelland I, Dahl AA, Haug TT, Neckelmann D (2002) The validity of the Hospital Anxiety and Depression Scale. An updated literature review. J Psychosom Res 52: 69–77. 11832252 10.1016/s0022-3999(01)00296-3

[20] Engum A (2007) The role of depression and anxiety in onset of diabetes in a large population-based study. J Psychosom Res 62: 31–38. 17188118 10.1016/j.jpsychores.2006.07.009

[21] Waitzfelder B, Gerzoff RB, Karter AJ, Crystal S, Bair MJ, Ettner SL, et al (2010) Correlates of depression among people with diabetes: The Translating Research Into Action for Diabetes (TRIAD) study. Prim Care Diabetes 4: 215–222. 10.1016/j.pcd.2010.07.002 20832375 PMC4269468

[22] Knudsen AK, Hotopf M, Skogen JC, Overland S, Mykletun A (2010) The health status of nonparticipants in a population-based health study: the Hordaland Health Study. Am J Epidemiol 172: 1306–1314. 10.1093/aje/kwq257 20843863

[23] Langhammer A, Krokstad S, Romundstad P, Heggland J, Holmen J (2012) The HUNT study: participation is associated with survival and depends on socioeconomic status, diseases and symptoms. BMC Med Res Methodol 12: 143 10.1186/1471-2288-12-143 22978749 PMC3512497

[24] Rubin RR, Knowler WC, Ma Y, Marrero DG, Edelstein SL, Walker EA, et al (2005) Depression symptoms and antidepressant medicine use in Diabetes Prevention Program participants. Diabetes Care 28: 830–837. 15793181 10.2337/diacare.28.4.830 PMC1314970

[25] Roy T, Lloyd CE, Pouwer F, Holt RI, Sartorius N (2012) Screening tools used for measuring depression among people with Type 1 and Type 2 diabetes: a systematic review. Diabet Med 29: 164–175. 10.1111/j.1464-5491.2011.03401.x 21824180

[26] Klungel OH, de Boer A, Paes AH, Seidell JC, Bakker A (1999) Cardiovascular diseases and risk factors in a population-based study in The Netherlands: agreement between questionnaire information and medical records. Neth J Med 55: 177–183. 10555434 10.1016/s0300-2977(99)00045-5

[27] Berge LI, Riise T, Fasmer OB, Lund A, Oedegaard KJ, Hundal O (2012) Risk of depression in diabetes is highest for young persons using oral anti-diabetic agents. Diabet Med 29: 509–514. 10.1111/j.1464-5491.2011.03530.x 22133020

[28] Knol MJ, Heerdink ER, Egberts AC, Geerlings MI, Gorter KJ, Numans ME, et al (2007) Depressive symptoms in subjects with diagnosed and undiagnosed type 2 diabetes. Psychosom Med 69: 300–305. 17470664 10.1097/PSY.0b013e31805f48b9

[29] Wahlqvist ML, Lee MS, Hsu CC, Chuang SY, Lee JT, Tsai HN (2012) Metformin-inclusive sulfonylurea therapy reduces the risk of Parkinson's disease occurring with Type 2 diabetes in a Taiwanese population cohort. Parkinsonism Relat Disord 18: 753–758. 10.1016/j.parkreldis.2012.03.010 22498320

[30] Hsu CC, Wahlqvist ML, Lee MS, Tsai HN (2011) Incidence of dementia is increased in type 2 diabetes and reduced by the use of sulfonylureas and metformin. J Alzheimers Dis 24: 485–493. 10.3233/JAD-2011-101524 21297276

[31] Collins MM, Corcoran P, Perry IJ (2009) Anxiety and depression symptoms in patients with diabetes. Diabet Med 26: 153–161. 10.1111/j.1464-5491.2008.02648.x 19236618

[32] Anstey KJ, Burns R, Butterworth P, Windsor TD, Christensen H, Sachdev P (2009) Cardiovascular risk factors and life events as antecedents of depressive symptoms in middle and early-old age: PATH Through Life Study. Psychosom Med 71: 937–943. 10.1097/PSY.0b013e3181beab60 19834045

[33] Hessler DM, Fisher L, Mullan JT, Glasgow RE, Masharani U (2011) Patient age: a neglected factor when considering disease management in adults with type 2 diabetes. Patient Educ Couns 85: 154–159. 10.1016/j.pec.2010.10.030 21112720 PMC3196056

[34] Li C, Ford ES, Strine TW, Mokdad AH (2008) Prevalence of depression among U.S. adults with diabetes: findings from the 2006 behavioral risk factor surveillance system. Diabetes Care 31: 105–107. 17934145 10.2337/dc07-1154

[35] Chen PC, Chan YT, Chen HF, Ko MC, Li CY (2013) Population-based cohort analyses of the bidirectional relationship between type 2 diabetes and depression. Diabetes Care 36: 376–382. 10.2337/dc12-0473 23150281 PMC3554286

[36] Lustman PJ, Anderson RJ, Freedland KE, de Groot M, Carney RM, Clouse RE (2000) Depression and poor glycemic control: a meta-analytic review of the literature. Diabetes Care 23: 934–942. 10895843 10.2337/diacare.23.7.934

[37] Mezuk B, Eaton WW, Albrecht S, Golden SH (2008) Depression and type 2 diabetes over the lifespan: a meta-analysis. Diabetes Care 31: 2383–2390. 10.2337/dc08-0985 19033418 PMC2584200

[38] Yoon JM, Cho EG, Lee HK, Park SM (2013) Antidepressant use and diabetes mellitus risk: a meta-analysis. Korean J Fam Med 34: 228–240. 10.4082/kjfm.2013.34.4.228 23904952 PMC3726790

[39] Barnard K, Peveler RC, Holt RI (2013) Antidepressant medication as a risk factor for type 2 diabetes and impaired glucose regulation: systematic review. Diabetes Care 36: 3337–3345. 10.2337/dc13-0560 24065841 PMC3781547

[40] Stringhini S, Batty GD, Bovet P, Shipley MJ, Marmot MG, Kumari M, et al (2013) Association of lifecourse socioeconomic status with chronic inflammation and type 2 diabetes risk: the Whitehall II prospective cohort study. PLoS Med 10: e1001479 10.1371/journal.pmed.1001479 23843750 PMC3699448

[41] Miller GE, Chen E, Parker KJ (2011) Psychological stress in childhood and susceptibility to the chronic diseases of aging: moving toward a model of behavioral and biological mechanisms. Psychol Bull 137: 959–997. 10.1037/a0024768 21787044 PMC3202072

[42] Berkowitz SA, Meigs JB, Wexler DJ (2013) Age at type 2 diabetes onset and glycaemic control: results from the National Health and Nutrition Examination Survey (NHANES) 2005–2010. Diabetologia 56: 2593–2600. 10.1007/s00125-013-3036-4 23995472 PMC3818392

