# Supplementary material for: Depression in Persons with Diabetes by Age and Antidiabetic Treatment: A Cross-Sectional Analysis with Data from the Hordaland Health Study
Source: PLoS One. 2015 May 26;10(5):e0127161. doi: 10.1371/journal.pone.0127161 (PMC4444007; doi:10.1371/journal.pone.0127161)
Supplement: S1 Table — (DOCX) [file pone.0127161.s001.docx]

**Supplementary table 1:** Associations of various measures of depression by antidiabetic treatment for persons aged 40-47 years in the Hordaland Health Study operationalizing diabetes as a 5 category variable.

| 40-47 years (n=18948) | *HADS-d≥8* | | *Antidepressant agents* | | *HADS-d≥8 and/or antidepressant agents* | |
| --- | --- | --- | --- | --- | --- | --- |
|  | n [%] | OR (95%CI)* | n [%] | OR (95% CI)* | n [%] | OR (95% CI)* |
| *No diabetes*  (n=18773) | 1795 (9.6) | 1 (ref) | 589 (3.1) | 1 (ref) | 2192 (11.7) | 1 (ref) |
| *Un-medicated diabetes*  (n=97) | 12 (12.4) | 1.35 (0.74, 2.48) | 7  (7.2) | 2.33 (1.07, 5.07) | 16  (16.5) | 1.50 (0.87, 2.56) |
| *Orally treated diabetes monotherapy*  (n=39) | 7  (17.9) | 1.99 (0.88, 4.51) | 5  (12.8) | 5.32 (2.05, 13.79) | 11 (28.2) | 2.95 (1.47, 5.94) |
| *Insulin and orally treated diabetes (n=3)* | 3  (100) | - | 0  (0.0) | - | 3  (100) | - |
| *Insulin treated diabetes* monotherapy (n=36) | 6  (16.7) | 1.87 (0.78, 4.50) | 1  (2.8) | 0.92 (0.13, 6.75) | 6  (16.7) | 1.51 (0.63, 3.63) |

*adjusted for gender.
